# Hegar–based method for aortic valve replacement in multiple valve surgery

**DOI:** 10.1186/s13019-018-0723-8

**Published:** 2018-05-21

**Authors:** Marco Gennari, Marco Agrifoglio, Gabriella Ricciardi, Gianluca Polvani

**Affiliations:** 1 IRCCS Centro Cardiologico Monzino, Via Parea 4, 20138 Milan, Italy; 2 Department of Cardiovascular Sciences and Community Health, University of Milan, Milan, Italy

**Keywords:** Mitral and aortic valve replacement, Tricuspid repair, Bivalvular replacement

## Abstract

**Background:**

Small aortic annuli are a challenge and a proper valve size is needed to avoid important prosthesis-patient mismatch, especially in case of multiple valves surgery.

**Case presentation:**

We proposed a technique involving the use of Hegar dilators in the aortic position while replacing the mitral valve, in order to maintain the proper aortic diameter. We used this method on two patients and we found it easy and reproducible.

**Conclusions:**

We report neither operative nor postoperative complications.

## Background

Small aortic annuli pose the challenge to correctly size the valve to avoid prosthesis-patient mismatch. Especially in the setting of double or triple valve surgery this condition may increase the risk. [1] We describe a method based on the use of Hegar dilators in the aortic position to maintain the proper aortic annulus diameter while performing mitral valve replacement.

## Case presentation

**Fig. 1:**
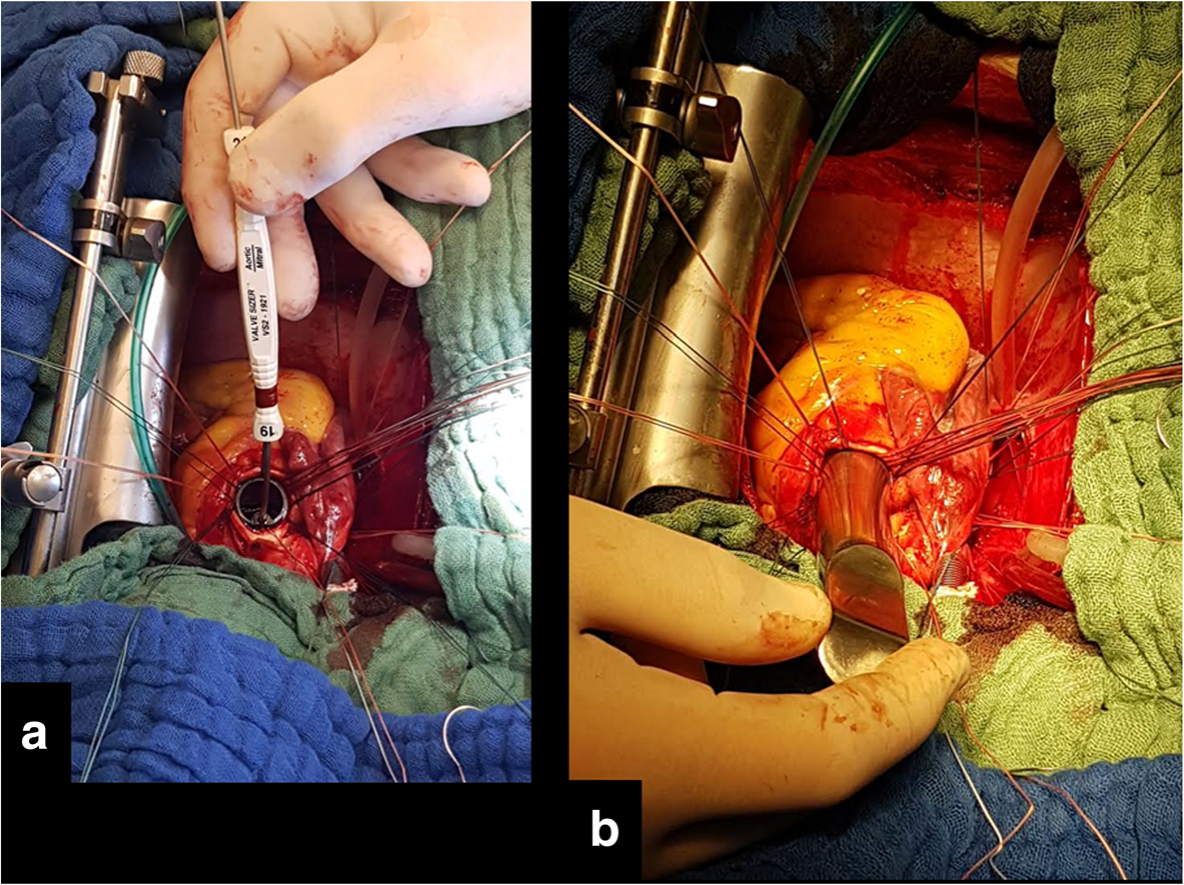
Intraoperative image of the aortic annulus sizing (**a**). The Hegar dilators is utilized to softly enlarge the aortic annulus (**b**)

**Fig. 2:**
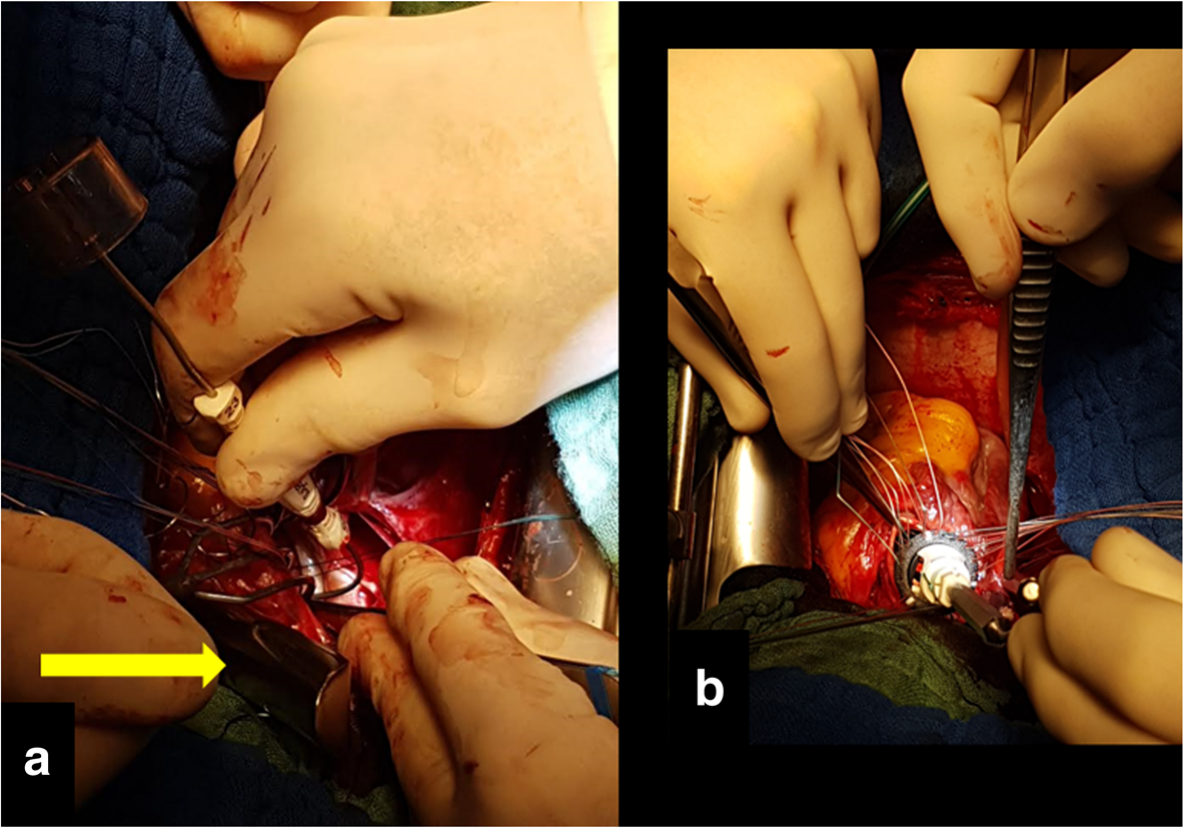
Intraoperative image. The mitral valve is measured and then fixed while the Hegar dilator maintains the correct aortic annulus diameter (**a**) and then the aortic valve prosthesis (**b**) is positioned in a supra-annular manner

In this setting we prefer peripheral cannulation; if tricuspid valve surgery is required a femoral-jugular cannulation is chosen. After cardiopulmonary bypass (CPB) institution and cardioplegic arrest of the heart the steps we follow are:

I. the aorta is transversally opened and the valve excised. The annular stitches are passed in an everting fashion to fit the valve in the supra-annular position
II. the left atrium is directly opened (or via the right atrium and the fossa ovalis if the tricuspid surgery is needed) and the mitral valve visualized and excised. The annular stitches are passed as usual
III. the aortic annulus is measured and generally for the small annuli the maximum measure achievable is 19 mm (Fig. 1 a). Then it is gently enlarged (Fig. 1 b) with the proper Hegar sizer (i.e. 20 -21 mm if the relative prosthesis sizer is 19 mm), avoiding any improper stress and thus reducing the risk of annular damage or dehiscence
IV. while the assistant keeps the Hegar within the aortic annulus (so above the left ventricular outflow tract in order to easily expose the mitral annulus), the mitral prosthesis is measured with the proper sizer, placed in the atrio-ventricular junction and tighten (Fig. 2 a)
V. then the Hegar dilator is removed and the aortic valve replaced as usual (Fig. 2 b)
VI. if tricuspid repair has to be performed the stitches are passed in the annulus *after* the mitral valve and tighten at the last

We performed this technique on two young women whom suffered from rheumatic multi valvular disease. The clinical and procedural features are listed in Table 1. We report neither procedural nor postoperative complications. The 1-year echocardiographic follow-up data showed a favorable outcome of the procedure (Table 2).

**Table 1:** Clinical and operative characteristics

Characteristics	# Patient 1	# Patient 2
Age	41 Years	39 Years
Bsa	1,46 m^2^	1,48 m^2^
Aortic annulus diameter	17 mm	17 mm
Tricuspid repair	Yes	No
Cardioplegia	Custudiol®	Custudiol®
Cpb time	192 min	171 min
Cross-clamp time	160 min	144 min
Aortic prosthesis diameter	19 mm	19 mm
Hegar aortic diameter	21 mm	20 mm
Mitral prosthesis diameter	25 mm	25 mm
Type of prosthesis	Biological	Mechanical
Tricuspid ring diameter	30 mm	/

*CPB* Cardio-Pulmonary Bypass, *BSA* Body Surface Area

**Table 2:** 1-year follow-up data

Characteristics	# Patient 1	# Patient 2
Mean ventriculo-aortic gradient	13 mmHg	12 mmHg
Mean aortic valve area	1,78 cm^2^	1,97 cm^2^
Ejection fraction	64%	66%
Mean transmitral gradient	4 mmHg	5 mmHg
Spap	29 mmHg	37 mmHg
Mean right atrio-ventricular gradient	4 mmHg	4 mmHg

*SPAP* Systolic Pulmonary Artery Pressure

## Discussion

**Fig. 3:**
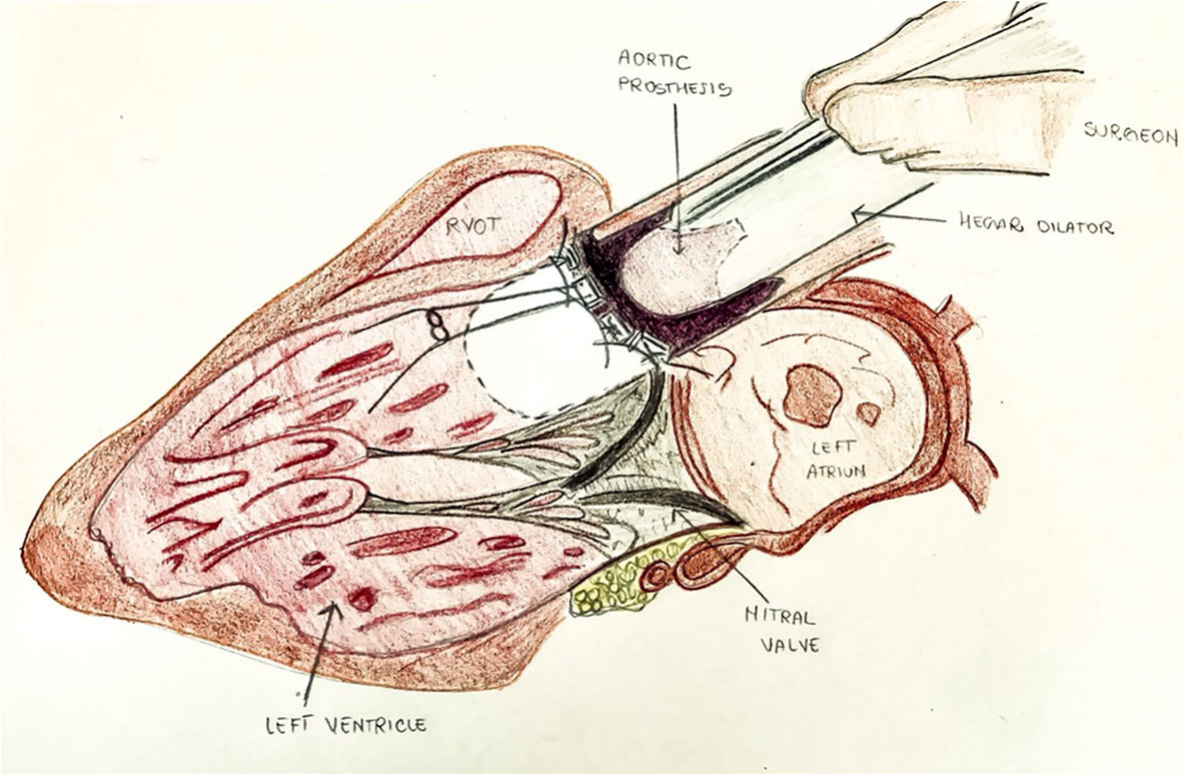
Drawning of long axis view showing in place Hegar dilator within the aortic root after suturing the aortic annulus and meanwhile suturing the mitral annulus

Small body surface area and small aortic annulus always pose a challenge, especially if additional valve replacement/repair is required. Because of the anatomical position of the aortic annulus – comprised between the central fibrous body of the heart and the conus ligament of the pulmonary valve – it is pleonastic that multiple valvular annular plications may reduce the effective diameter and thus lead to an undersized aortic valve prosthesis and a severe prosthesis-patient mismatch may occur (Fig. 3).

We describe this simple and reproducible method to maintain the proper aortic diameter while suturing and fixing the mitral prosthesis.

A word of caution must be said in the case of advanced rheumatic disease causing annular calcification and stiffness; in this case we suggest to do not force the Hegar diameter above 1 mm over the valvular tester measure.

Calafiore et al. [2] described another modified sequence of valvular preparation and fixation in case of small annulus that implies the removal of both the valves and the fixation of mitral prosthesis first and then the aortic one.

We decided to fix the mitral prosthesis first while maintaining the right aortic annular shape because we found very challenging to correctly tight the valve in the antero-lateral portion of the annulus once the aortic prosthesis is placed.

## Conclusion

We report no complications in using this method. We believe that this could be useful in the setting of very small aortic annuli and concomitant mitral valve surgery.

## Abbreviation

CPB: Cardio-pulmonary bypass
